# Comment on “Bioactive glass S53P4 vs. autologous bone graft for filling defects in patients with chronic osteomyelitis and infected non-unions – a single center experience” by Steinhausen et al. (2021)

**DOI:** 10.5194/jbji-6-199-2021

**Published:** 2021-05-27

**Authors:** Martin McNally

**Affiliations:** Oxford Bone Infection Unit, Nuffield Orthopaedic Centre, Oxford University Hospitals, Oxford, UK

I read with interest the recent paper by Steinhausen et al. (2021). This paper reports the outcome of a retrospective review of bioactive glass compared to autologous bone graft (ABG) in a staged surgical protocol for treating established bone infection. The authors claim that bioglass is equally effective as ABG in eradicating infection but acknowledge that their evidence is weak.

The surgical treatment of chronic bone infection has been reported for well over a century. In 1931, Jacob Kulowski published his classical series of 130 cases of osteomyelitis, treated by Orr's method (Kulowski, 1931). His surgical debridement technique was similar to that described by Steinhausen et al. (2021), and he achieved a recurrence rate of 24 % (mean follow-up: 19 months), in the pre-antibiotic era, with no defect fillers. In this new series of 83 patients, the recurrence rate with bioglass was 29 % and 19 % for ABG. Clearly the addition of bioglass or bone graft has not improved outcome.

The authors state that the longer follow-up period for the ABG group is not a source of bias because “most complications occurred within 12 months”. This same group previously published 50 of their 51 bioglass cases (Malat et al., 2018) with a mean follow-up of 12.3 months and a recurrence rate of 14 %. This has increased to 29 % with a longer follow-up (mean 20.5 months). It would appear that the recurrence rate doubled in the 8 months after the first year.

There are no large randomized trials directly comparing the use of bioglass with other options, and the authors correctly report that outcomes of small studies are inconclusive. However, there are two high-quality animal studies (Xie et al., 2009; Boot et al., 2020). In a rabbit model of MRSA osteomyelitis, Xie et al. (2009) reported that bioglass was no more effective than debridement alone (success rate 36 % for debridement and 18 % for debridement and bioglass) but the addition of local antibiotics to calcium sulfate or bioglass increased success to 73 % and 81 % respectively. Boot et al. (2020) studied the effect of hydrogel and bioglass in an infected tibial nail model. They showed that bioglass or hydrogel without antibiotics produced no reduction in infection, but vancomycin-loaded hydrogel could eradicate established infection.

It has been widely claimed that bioglass has the advantage of reducing the risk of antimicrobial resistance. This paper shows that cases treated with bioglass required significant additional systemic antimicrobial therapy after repeated surgery or recurrence. It has been shown that repeated surgery promotes change in bacterial cultures (Rupp et al., 2020). Recurrent, and often suboptimal, systemic therapy drives selection pressure and multi-drug resistance. There is no evidence that local antimicrobial therapy contributes to this. The recent study of Bidossi et al. (2020) demonstrated that prolonged exposure to high-dose local antibiotics in a ceramic carrier was not associated with any adaptations in bacteria producing antimicrobial resistance. In contrast to bioglass, the use of local antimicrobials may allow reduced systemic therapy and better antibiotic stewardship (Masrouha et al., 2018; Dudareva et al., 2019).

Steinhausen et al. (2021) also claim that “The tolerability of BAG is even described as superior when compared to other bone substitutes”. We cannot find any evidence for this statement in their paper. When comparing to the paper they quote as evidence (McNally et al., 2016), the claim is not supported. If we compare recurrence rate, reoperation rate, failure of bone healing and amputation rate between the two papers, they report 29 %, 47 %, 23 % and 6 % respectively. In our study of 100 cases, the figures were 4 %, 3 %, 13 % and 0 %. We believe these outcomes would be more tolerable for patients.

Perhaps the most important conclusion from this paper is that serial debridement, followed by defect filling without local antimicrobials, is not a very effective treatment. We would not recommend use of a hip or knee prosthesis with a 29 % failure rate within 2 years. This problem was identified in the 1970s and prompted the use of antibiotic-loaded PMMA beads by Klemm and Buchholz (Klemm, 1993). There is now encouraging evidence around the use of single-stage surgery, facilitated by modern local antibiotic carriers, with few complications and high infection eradication rates, even in the most complex cases (Masrouha et al., 2018; McNally et al., 2016; Ferguson et al., 2014; Lam et al., 2019; Pincher et al., 2019; Lorentzen et al., 2020; Drampalos et al., 2018; Jiang et al., 2020; Pesch et al., 2020; Hutting et al., 2021; Mifsud et al., 2020; Zhou et al., 2021).

## Data Availability

All data pertaining to this letter is included in the text.
